# Young Adult Caregiving Daughters and Diagnosed Mothers Navigating Breast Cancer Together: Open and Avoidant Communication and Psychosocial Outcomes

**DOI:** 10.3390/cancers15153864

**Published:** 2023-07-29

**Authors:** Carla L. Fisher, Gemme Campbell-Salome, Diliara Bagautdinova, Kevin B. Wright, Larry F. Forthun, Kelsey C. Bacharz, M. Devyn Mullis, Bianca Wolf, Deidre B. Pereira, Lisa Spiguel, Carma L. Bylund

**Affiliations:** 1 Department of Health Outcomes and Biomedical Informatics, College of Medicine, University of Florida, Gainesville, FL 32610, USA; mullis.md@ufl.edu (M.D.M.); carma.bylund@ufl.edu (C.L.B.); 2 Geisinger Medical Center, Danville, PA 17822, USA; gcampbell3@geisinger.edu; 3 Department of Advertising, College of Journalism and Communications, University of Florida, Gainesville, FL 32611, USA; dbagautdinova@ufl.edu; 4 Department of Communication, College of Humanities and Social Sciences, George Mason University, Fairfax, VA 22030, USA; kwrigh16@gmu.edu; 5 Department of Family, Youth and Community Sciences, University of Florida, Gainesville, FL 32611, USA; lforthun@ufl.edu; 6 Department of Clinical and Health Psychology, College of Public Health and Health Professions, University of Florida, Gainesville, FL 32610, USA; kbacharz@phhp.ufl.edu (K.C.B.); dpereira@phhp.ufl.edu (D.B.P.); 7 Department of Communication Studies, University of Puget Sound, Tacoma, WA 98416, USA; bwolf@pugetsound.edu; 8 Department of Surgery, College of Medicine, University of Florida, Gainesville, FL 32610, USA; lisa.spiguel@surgery.ufl.edu

**Keywords:** mother, daughter, young adult caregiver, breast cancer, openness, avoidance, communication, distress

## Abstract

**Simple Summary:**

Breast cancer is a shared experience for diagnosed mothers and their young adult caregiving daughters (YACDs). They struggle to talk about cancer and receive no guidance for navigating challenging but critical care conversations. Daughters in young adulthood also tend to avoid cancer-related talk, which is associated with poorer biopsychosocial outcomes, whereas openness between mothers and daughters is tied to better biopsychosocial outcomes. We sought to determine mothers’ and YACDs’ most challenging topics as well as their preferred strategies that help them engage in these discussions, while also exploring associations between openness/avoidance and their psychosocial outcomes. Results highlight how mother–daughter communication approaches (i.e., avoidance and openness) intersect with their psychological distress and relational satisfaction. Collectively, findings demonstrate that if we are to promote better psychosocial outcomes for diagnosed mothers and their caregiving daughters, we must recognize the influential role their communication plays in their well-being.

**Abstract:**

For many diagnosed mothers and their daughters, breast cancer is a shared experience. However, they struggle to talk about cancer. This is particularly true when the daughter is in adolescence or young adulthood, as they tend to be more avoidant, which is associated with poorer biopsychosocial outcomes. When daughters are their mother’s caregivers, daughters’ burden and distress are heightened. Young adult caregiving daughters (YACDs) are the second most common family caregiver and encounter more distress and burden than other caregiver types. Yet, YACDs and their diagnosed mothers receive no guidance on how to talk about cancer. Thirty-nine mother/YACD pairs participated in an online survey to identify challenging topics and strategies for talking about cancer, and to explore associations between openness/avoidance and psychosocial outcomes. YACDs and mothers reported the same challenging topics (death, treatment-related issues, negative emotions, relational challenges, YACDs’ disease risk) but differed on why they avoided the topic. YACDs and mothers identified the same helpful approaches to navigate conversations (openness, staying positive, third-party involvement, avoidance). Avoidance was correlated with more distress whereas openness was correlated with better psychosocial outcomes. These results provide a psychosocial map for a mother-YACD communication skills intervention, which is key to promoting healthy outcomes.

## 1. Introduction

More than 3.8 million women in the United States live with breast cancer, and more than 13% of women will be diagnosed at some point in their life [1]. For many diagnosed women, breast cancer is a shared experience with their daughters [2,3,4,5,6]. They cope with the disease together, exchanging critical social support [3,7,8]. This is true even for younger daughters, in adolescence through young adulthood (AYAs) aged 15–39, as they shift to a more supportive role for their mother after diagnosis, often for the first time in their relational history [3,4,5,6]. AYAs report wanting to emotionally and physically “be there” for their mother [4], and mothers emphasize that their daughter’s support is integral to their care, coping, and well-being [2,3,4,5,6,7,8].

Coping together, however, is not without challenges for mothers and daughters, particularly AYAs. AYA daughters struggle in their new role while also trying to manage their own distress and coping needs [9]. Diagnosed mothers and daughters also mirror one another in negative psychological and physiological effects [4,5,6,10,11,12,13,14,15,16]. When diagnosed mothers report posttraumatic stress symptoms, increased stress hormones such as cortisol, and/or decreased immunological functioning, their daughters do as well [10,17]. Diagnosed mothers and their daughters also describe grappling with distressing “psychological chronic risk”, in which they simultaneously fear mothers’ disease recurrence while worrying about their daughters’ future risk [12].

Daughters serving in a caregiving capacity for their mothers experience additional role-related burdens and stress. Young adult caregiving daughters (YACDs) aged 18–39 are the second most common type of family caregiver after spouses [2,18,19] and comprise approximately one-third of caregivers for adult women diagnosed with breast cancer [2,18,19,20,21,22]. Caregiving significantly affects YACDs’ socioemotional well-being [23,24,25]. Compared to older adult child caregivers as well as spousal caregivers, YACDs report higher levels of depression, caregiving distress, and burden, and face a higher risk of developing socioemotional or behavioral problems [26,27,28].

Despite mothers’ and YACDs’ shared breast cancer experience and interrelated health outcomes, they remain unsupported and underrepresented in cancer caregiving research. Research informing the Family Systems Illness Model has elucidated the need for both family-centered cancer interventions—to fully attend to diagnosed parents’ and their children’s needs—and developmentally targeted interventions [29,30,31]. In other words, diagnosed parents’ needs and their children’s needs are distinct and informed by both family system factors (e.g., relational role) and lifespan factors (e.g., developmental phase in life) [32,33]. YACDs’ and their mothers’ cancer-related needs after diagnosis are ultimately embedded within complex intergenerational relational dynamics tied to variant developmental needs, hierarchically informed relational roles, and sociohistorical (generational) differences in talking about health [32,34].

Additionally, the Cancer Family Caregiving Experience Model identifies communication in the family system and in clinical settings as mechanisms that affect diagnosed individuals’ and their caregivers’ coping behavior and related health outcomes [3,35,36,37,38,39]. While limited, interventions to improve caregivers’ communication skills show better caregiver and patient health outcomes [16,40,41,42,43]. Yet, such interventions typically do not give directions on how or what to discuss and typically target spouses [44]. Research is needed to inform communication skills interventions that support the psychosocial needs of diagnosed mothers and their YACDs—the most distressed group of family caregivers who will also face their own personal risk of the disease in the future.

### Mother–Daughter Communication and Breast Cancer Coping and Caregiving

Mothers and AYA daughters, especially YACDs, need communication skills interventions to navigate breast cancer. They not only describe challenging relational dynamics, but they also engage in maladaptive communication patterns [2,5,10,12,17,45,46,47,48,49,50]. Unlike older mother-adult daughter age groups, AYA daughters consistently engage in avoidant behavior, withdrawing from or avoiding cancer-related communication with their mothers, which contributes to both AYAs’ and their mother’s distress [4,5,43,51,52,53,54,55]. While limited, research has shown that AYAs and their mothers struggle with talking about daughters’ future breast cancer risk, negative affect like feelings of sadness, and mothers’ clinical experiences particularly physical changes after a mastectomy [51]. Mothers also describe avoiding or withholding information to shield their AYAs from distress [4,5,54,56]. Yet, mothers’ avoidant coping is linked with poorer health outcomes, and AYA daughters describe their mother’s withholding as stress-inducing [4,5,51].

In contrast to avoidant behavior, open communication about cancer (i.e., comfort with and engagement in both breadth and depth of talk) is associated with better disease adjustment and health outcomes for diagnosed mothers and their daughters [57,58,59]. Diagnosed mothers who report more openness in their mother–daughter relationship also report better relational well-being and physical health outcomes [56]. The benefits of open communication in parent–child bonds after a cancer diagnosis may even extend to better clinical communication. For instance, when midlife adult child caregivers report more open communication with their parent diagnosed with a blood cancer, they also report better clinical communication experiences with their parent’s clinicians [60]. While open communication may promote better biopsychosocial outcomes and disease adjustment both at home and in the clinic, studies informing the Family Communication Patterns Theory (FCP) have demonstrated that family relationships develop patterns or norms of communicating over their relational history that are characterized, in part, by how openly (or not) they communicate [61]. These patterns will inevitably inform how they cope with strenuous experiences like a breast cancer diagnosis. Facilitating openness after a cancer diagnosis may be especially challenging for parent–child bonds (such as mothers and daughters) who do not typically communicate openly with one another (i.e., do not have a relational history of open communication).

Unfortunately, diagnosed parents and their adult, young adult, or adolescent child caregivers do not receive guidance on how to talk about cancer as they cope together, which may be especially needed for family relationships that are not characterized by open communication patterns. How topics are discussed, and the respective psychological well-being (e.g., distress state) of both mothers and daughters during such conversations, likely inform whether mothers and AYAs engage in openness, as their behavior may contribute to adverse health outcomes [16,56,62]. For example, observational data shows that diagnosed mothers’ denial, hostility, or antisocial behavior when discussing breast cancer is associated with AYA daughters’ increased cortisol levels [16]. Additionally, AYAs’ whining and complaining behaviors during such conversations are linked to their increased cortisol [16]. While mothers and AYA daughters may be receptive to talking, the way they discuss cancer has implications for their coping and health outcomes.

A recent study identified several strategies both AYAs and their diagnosed mothers perceive as helpful when having breast cancer-related discussions, including having mothers initiate the conversation, keeping the focus positive, and limiting the conversations [55]. However, no studies have examined AYAs who self-identify as caregivers of their diagnosed mothers (i.e., YACDs) although they are increasingly serving as mothers’ caregivers and are at a higher risk of burden and distress [21,22,23,24,25,41,46,47,48,63]. Research demonstrates that even interventions that broadly address communication (e.g., encouraging openness versus avoidance) positively impact outcomes [64,65]. Mothers and their YACDs may experience better disease adjustment and reduced distress from a targeted communication skills intervention that helps them identify challenging but important care issues while teaching them how to communicate in health-promoting ways.

In order to develop such an intervention, we explored four aims: (1) To identify the most challenging cancer-related topics and examine relationships with mothers’ and YACDs’ cancer-related topic avoidance and avoidant coping; (2) to explore relationships between mothers’ and YACDs’ communication outcomes (topic avoidance and open communication) with two psychosocial outcomes (distress and relational satisfaction); (3) to explore relationships between mothers’ and YACDs’ openness and two communication outcomes (topic avoidance and mothers’ clinical communication skills); and (4) to identify strategies for talking about cancer care related issues.

## 2. Materials and Methods

We recruited YACDs and their diagnosed mothers to participate in an online survey about their communication dynamics and psychosocial outcomes. To be eligible, YACDs had to be (1) female, (2) aged 18–39, (3) a daughter of a female breast cancer patient in treatment (defined as radiation, chemotherapy, or surgical), and had to (4) identify as a caregiver. Eligible mothers had to be (1) female, (2) diagnosed with breast cancer, (3) in treatment, and had to (4) have a YACD.

### 2.1. Recruitment

Diagnosed mothers and YACDs were recruited from November 2020–November 2021 using multiple strategies: (1) in-person clinical recruitment in a cancer center by screening eligible mothers at appointments; (2) flyers posted on clinic screens, bulletin boards, in waiting rooms, and in information folders disseminated by a breast cancer navigator; (3) a patient research registry, maintained by the University of Florida Health system; (4) ResearchMatch, a national registry of volunteers that was developed by academic institutions and supported by the U.S. National Institutes of Health as part of the Clinical Translational Science Award program; and (5) social media dissemination with advocacy groups (e.g., FORCE). Recruitment materials included an email and phone number that interested women could contact to screen for eligibility. To maximize recruitment, we asked women for their mother/YACD’s information.

### 2.2. Online Survey

Mothers and YACDs received a code upon enrollment to link dyads. Survey items were drawn from validated and reliable scales to assess communication and psychosocial outcomes. Two open-ended items captured challenging and potentially avoided cancer-related topics (for Aim 1: “What are the most challenging topics for you and your mother/daughter to discuss?”) as well as helpful strategies to discuss these topics (for Aim 2: “What has helped you talk about challenging topics with your mother/daughter?”).

### 2.3. Measures

#### 2.3.1. Cancer Topic Avoidance

The Communication Topic Avoidance (CTA) scale (42 items) was used to identify topics they avoid talking about [66,67]. The measure captures avoidance of seven cancer-related topic areas: death, treatment, risk, burden, feeling, relating, and health care issues. Mothers and YACDs rated on a 5-point Likert-type scale how much they “avoid talking to my mother/YACD about [topic]”. The reliability coefficient was α = 0.97 for both mothers (M = 2.8; SD = 0.8) and YACDs (M = 3.0; SD = 0.09).

#### 2.3.2. Avoidance

The Avoidance subscale (8 items) of the Impact of Events Scale (IES-R) measured women’s avoidance [61]. This subscale captures avoidance when coping with a traumatic event (such as breast cancer) and asks women to report their avoidant coping (e.g., “I tried not to think about it”; “I tried not to talk about it”) within the past 7 days Mothers and YACDs rated on a 5-point Likert-type scale their avoidance ranging from “not at all = 0” to “extremely = 4”. The reliability coefficient was α = 0.92 for mothers (M = 3.2; SD = 0.6) and α = 0.90 for YACDs (M = 3.4; SD = 1.0).

#### 2.3.3. Mother–Daughter Open Communication

The short-form Revised Family Communication Pattern (RFCP) scale (6 items) associated with FCP theory was used to measure how open mothers and daughters typically communicate in their relationship [68]. Using a 7-point Likert-type scale (strongly disagree = 1 and strongly agree = 7) mother and YACDs rated their relational communication pattern (e.g., “I can tell my mother/YACD almost anything”; “I really enjoy talking to my mother/YACD, even when we disagree”). The reliability coefficient was α = 0.84 for both mothers (M = 4.3; SD = 0.6) and YACDs (M = 4.1; SD = 0.8).

#### 2.3.4. Clinical Communication Skills

The Patient Report of Communication Behavior (PRCB) [69] (11 items) was used to measure mothers’ perceptions of their communication skills when talking to their clinicians. Mothers rated their current clinical communication skills (e.g., “I tell my doctors when I want more information about something”) on a 5-point Likert-type scale (never = 1 to always = 5). The scale was reliable (α = 0.94, M = 4.2, SD = 0.08).

#### 2.3.5. Psychological Well-Being

Distress was measured using the Profile of Moods State (POMS) short form–cancer (37 items), which has been utilized to measure cancer caregivers’ distress [70,71]. Using a 5-point Likert-type scale (not at all = 0 and extremely = 4), mothers and YACDs rated their feelings during the past week (e.g., “tense,” “angry,” “discouraged”). Items were then grouped into distress state scores for vigor, depression, anger, tension, confusion, and fatigue (see Section 3.2.2 for descriptive statistics for each mood state). The various mood states were combined into a composite score as a measure of the overall distress state. The reliability coefficient for all items was α = 0.98 for mothers (M = 70.8; SD = 28.1) and α = 0.97 for YACDs (M = 66.6; SD = 25.8).

#### 2.3.6. Relational Satisfaction

The Modified Marital Opinion Questionnaire (MOQ) (11 items) is a relational satisfaction scale that was adapted to refer to mothers and YACDs [72]. They rated their relational well-being using a 7-point semantic differential scale (e.g., “rewarding/disappointing” and “miserable/enjoyable”). The reliability coefficient for all items was α = 0.93 (M = 5.8; SD = 1.1) for mothers and α = 0.92 (M = 5.1; SD = 1.2) for YACDs.

### 2.4. Data Analysis

To address our first and last aims (avoided topics and strategies for navigating topics), we conducted a thematic analysis on two open-ended items. To promote rigor, multiple authors (coders) with qualitative analysis training were involved in thematically analyzing the open-ended responses using a constant comparative method (CCM) approach [73,74,75]. CCM includes immersing oneself in the data by reading all responses, using inductive (i.e., open) and/or deductive (i.e., closed) coding to identify patterns in responses, and assigning labels (i.e., codes), collapsing patterns into categories or themes, and identifying rich responses for the presentation of thematic findings [73,74]. An inductive and deductive analysis was conducted by three authors, with analyses kept separate by group (YACDs versus mothers) to triangulate findings to identify similarities and differences in experiences. First, one author (MDM) conducted an inductive analysis of all responses with the senior author (CLF) reviewing all data analyzed and associated with each theme to validate findings. CLF also used deductive coding to collapse any findings into categories that were identified as challenging topics in the survey measure (CTA scale) to refine and establish a final codebook. A third author (DB) then used the codebook to deductively analyze the data to validate the analysis for each perspective (YACD and mothers), further ensuring rigor. Frequencies were determined to ensure saturation given all respondents received the same question [76].

To explore Aims 1-3, scale data were analyzed using SPSS version 26. Scores for both YACDs and their mothers were calculated on all measures, and each set was used in the t-test and correlation analyses reported below. A series of related measures t-tests were used to compare differences in topic avoidance scores between YACDs and their mothers. All other analyses were conducted using bivariate Pearson’s correlation tests.

## 3. Results

### 3.1. Participants

A total of 39 mother–YACD dyads participated in the online survey; an additional 5 mothers whose YACD did not complete the study also participated. The mean age for mothers was 51 (SD = 10.3) and mean age for YACDs 23.3 (SD = 5.3). See Table 1 for additional demographic information.

### 3.2. Topic Avoidance and Avoidant Coping

#### 3.2.1. Qualitative Findings

Aim 1 explored cancer-related topics mothers and YACDs avoid and find challenging to discuss. Mothers and YACDs in all 39 dyads responded to the open-ended item addressing topics that were most challenging to discuss. An additional 5 mothers’ responses were included whose YACD did not complete the study. YACDs’ responses were longer on average (1–230 words, M = 35.69, SD = 45.45) than mothers’ (1–332, M = 33.75, SD = 51.54), and the range of mothers’ responses was larger than YACDs’. While both YACDS and mothers reported the same five topics as challenging (death, treatment-related issues, negative emotions, relational challenges or relating, YACDs’ disease risk), they differed on why they avoided the topic or found it challenging.

##### Death

Addressing mortality was the most challenging topic reported (26 mothers, 20 YACDs). Both groups described each other, as well as themselves, as avoidant of this topic. They explained their avoidance as a way to shield each other from distress, believing that talking about mortality would “upset” them, make them “sad”, or “deprive [mother/daughter] of hope”. YACDs emphasized focusing on the present: “I do not want to talk about death. I am aware that it will happen but refuse to let it steal time from the present by acknowledging it” (YACD-11, age 24). YACDs’ responses also reflected a fear of their mother’s death whereas mothers’ responses illustrated a fear for their YACD’s welfare if they die: “The fear of death has been a very big challenging topic for me. … I feel like leaving this poor girl in this world is not something I want to do” (Mother-31, age 45).

##### Treatment-Related Issues

YACDs and mothers described challenges addressing “certain aspects of treatment” (14 mothers, 12 YACDs). For YACDs, they focused on their mother’s avoidant behavior, which inhibited their ability to provide care:

*[My mom’s] pain is hard because she disconnects so quickly. Self-advocating is hard to discuss because it is very emotionally charged, and I don’t want to take her autonomy away. … Her frustration with her new level of functioning and chronic pain with the push back I get from her in lightening her load mentally/physically while she recovers (YACD-6, age 28).*

Though YACDs did not describe challenges discussing their mothers’ physical changes, mothers perceived that YACDs found this treatment topic challenging: “The change in my physical appearance due to the disease and the treatments because I know that it affects her a lot” (Mother-19, age 52).

##### Negative Emotions

Mothers and YACDs struggled to disclose or discuss their fears, sadness, and anger (13 mothers, 9 YACDs). Mothers wrote about not being able to share “the negative feelings I have in mind,” noting YACDs’ discomfort: “She always seems stressed or overwhelmed. She can never just sit and relax. I think a lot of this has to do with her being scared of losing me or not having all the answers” (Mother-29, age 67). Interestingly, YACDs also expressed challenges sharing their feelings and perceived that their mother was not willing to hear such disclosures: “She is so concerned with her current situation (understandably) that she doesn’t really care about my situation and or feelings. She gets irritated when I bring up what makes me unhappy or how I’m stressed and even depressed” (YACD-44, age 21).

##### Relational Challenges or Relating

YACDs and their mothers avoided relational talk (6 mothers, 5 YACDs). Mothers avoided discussions about their relationship as well as their YACDs’ personal relationships. Relational challenges were associated with a feeling of disconnection: “I feel like our relationship has been affected. [Daughter] always seems stressed or overwhelmed” (Mother-29, age 67). YACDs also avoided discussing relational challenges but described doing so to prioritize their mother’s needs: “It is really challenging for us to discuss our relationship. I know that [my mother’s] going through a lot right now, so I find myself often holding my tongue when I’d rather be setting a boundary or communicating my feelings” (YACD-43, age 19).

##### YACDs’ Disease Risk

Although less commonly reported, YACDs and mothers found risk discussions challenging, which was heightened for elevated-risk families (3 mothers, 2 YACDs). YACDs shared that they did not want to think about risk this early in adulthood, which was also linked with not being ready to make risk-management decisions:

*It is challenging because we just recently found out breast cancer runs in our family and the strong likelihood I will get it. This has brought up the fact about having kids before I get cancer, the fear she has that I will be scared to have kids and the fact that if I wait long enough I may not get the chance. As I have not been in a long relationship, this seems far off in my future, and that pressure to move faster is hard on me. (YACD-47, age 24)*

Mothers admitted feeling distressed about their YACD’s risk: “The fact that she may carry the same genetic mutations and whether she wants to find out at her young age. I really don’t want her to do anything too soon that would not allow her to have children” (Mother-43, age 60).

#### 3.2.2. Quantitative Results

Statistical analyses validated and extended qualitative findings for the first aim by further capturing differences between mothers’ and YACDs’ perceptions (see Table 2). Like their open-ended responses, mothers and YACDs rated death as the most avoided topic. However, they also avoided discussing care burden—a topic that was not captured in their open-ended responses. Additionally, a series of related measures t-tests revealed that mothers and YACDs only differed significantly in terms of avoiding talking about death, with YACDs (M = 3.56; SD = 0.94) having significantly higher avoidance than mothers (M = 3.23; SD = 1.13, t = −2.156, *p* = 0.03). All other differences were nonsignificant.

For Aim 1, we also investigated the relationship between the degree of mothers’ and YACDs’ individual avoidant coping responses and their avoidance of cancer-related topics. Results indicate that mothers’ avoidant coping positively correlated with their topic avoidance of four of the challenging topics: death, treatment, burden, and healthcare. In contrast, YACDs’ avoidant coping was positively correlated with their avoidance of all topics (see Table 3).

### 3.3. Avoidance and Psychosocial Outcomes

#### 3.3.1. Distress

Table 4 and Table 5 report the bivariate correlations for mothers’ and YACDs’ overall topic avoidance scores and distress (Aim 2). For both mothers and YACDs, topic avoidance was positively associated with greater anger. Mothers’ topic avoidance was also positively associated with increased depression, tension, confusion, and fatigue scores. Additionally, while mothers’ topic avoidance was not significantly correlated with vigor, the correlation analysis revealed that lower YACD avoidance scores were significantly correlated with increased vigor scores (a positive mood state).

#### 3.3.2. Relational Satisfaction

YACDs’ and mothers’ topic avoidance and relational satisfaction were also related (Aim 2). A correlation analysis indicated that higher YACDs’ topic avoidance scores were significantly correlated with lower relational satisfaction scores for mothers, r (N = 39) = −0.52, *p* < 0.001. Additionally, a correlation analysis indicated that higher topic avoidance scores for mothers were correlated with lower relational satisfaction scores for YACDs (M = 5.14; SD = 1.22), r = −0.49, *p* < 0.001.

### 3.4. Open Communication and Topic Avoidance

For Aim 3, we investigated mothers’ and YACDs’ openness is tied to their cancer-related topic avoidance. A correlation analysis showed that increased mother–YACD openness scores were inversely related to avoiding the topics of burden and relating for mothers, and they were inversely related to avoiding the topics of death and relating for YACDs (see Table 6).

### 3.5. Open Communication and Clinical Communication Skills

For Aim 3 we also examined associations between mothers’ and YACDs’ openness and mothers’ perceptions of their clinical communication skills. A correlation analysis showed that YACDs’ reports of more open mother–YACD communication (M = 4.05; SD = 0.78) were significantly correlated with mothers’ perceptions of having better clinical communication skills (PRCB measure) (M = 4.21; SD = 0.84), r (N = 39) = 0.39, *p* = 0.012. However, mothers’ perceptions of openness with YACDs were not associated with better perceptions of their own clinical communication.

### 3.6. Strategies for Navigating Challenging Topics

To answer Aim 4, 43 mothers and 40 YACDs responded to an open-ended item that asked them to identify what helped them talk about challenging cancer-related topics. YACDs’ responses were longer (2–110, M = 32, SD = 27.33) than mothers’ (2–75, M = 24.25, SD = 16.38). Both mothers and their YACDs reported four approaches they perceived helped them navigate these conversations: (1) valuing openness, (2) staying positive, (3) third-party involvement, and (4) avoidance. Slight differences were noted in their perceptions of the strategy as well as their identification of one strategy described below.

#### 3.6.1. Talking Openly

YACDs and mothers stressed the importance of being open or “not hold[ing] it in” (18 mothers, 16 YACDs). Although both acknowledged discomfort talking openly, mothers perceived their YACD’s willingness to talk helped them feel supported. Additionally, mothers perceived openness was a way to ensure YACDs were informed: “I know that most topics are really challenging to talk about, but I feel like my daughter should be aware of it so that mindset has helped me talk about it with her” (Mother-15, age 40). YACDs emphasized openness as a way to facilitate honesty: “When my mom first got diagnosed and we found out about all the surgeries, I told her that I always wanted her to be honest with me about what was going on” (YACD-3, age 18). YACDs also shared how their openness was needed to counteract mothers’ avoidant behavior:

*Being persistent and listening as much as I can. She feels so devalued by the medical system so consistently showing her I will listen has built up trust so we can talk about the hard things. This is something I work on with every conversation because she is so quick to try and hold everything herself. I can sit in the logical side when emotions are too much or I can just sit in the pain with her knowing not a word I could ever say will fix it. So, I suppose straightforward questions about how she would like to be supported that day or during that conversation. (YACD-6, age 28)*

#### 3.6.2. Staying Positive

Mothers and YACDs valued positivity (8 mothers, 7 YACDs). Mothers focused on YACD’s positive outlook: “Her optimism helps me to keep fighting” (Mother-16, age 62). Similarly, YACDs stressed the importance of positive communication like positively reframing things and using humor: “Laughter! I can’t stress this enough. Me and my mom laugh all the time about the stupidest stuff, and it helps take away the pain you’re feeling at the moment! I promise this works” (YACD-10, age 20).

#### 3.6.3. Third-Party Involvement

While both mothers and YACDs sought third-party assistance, YACDs reported this approach more (2 mothers, 8 YACDs). Outside sources helped YACDs communicate with their mothers, which included their “therapist”, “family doctor”, “social media”, and other family members (e.g., grandmother). Mothers also wrote about getting advice from friends and breast cancer support groups: “My breast cancer support group helps me create ways to discuss sensitive topics with her” (Mother-13, age 43).

#### 3.6.4. Avoidance

Interestingly, both mothers and YACDs responded that avoidance was helpful (4 mothers, 8 YACDs). At times, avoidant behavior was reciprocal, as this YACD shared:

*But when [my mother] makes it clear to me that she does not wish to talk further, I walk away and don’t bring it up again until she appears ready to do so (i.e., she brings it up, or a third-party like a doctor brings it up). (YACD-1, age 39)*

Mothers perceived avoidance buffered their YACD from distress: “I feel like keeping the pain and discomfort from her is better than placing that burden and concern upon her” (M-2, age 55). Avoidance was also used as a final resort tactic, as this YACD illustrated: “Nothing has really helped. I simply zone most of the challenging conversations out because I don’t want to think about it. We really need a better way to communicate. It just always seems so negative” (YACD-51, age 22).

## 4. Discussion

Young adult family cancer caregivers, like YACDs, have been labeled the “hidden” generation of caregivers given their absence in both policy and resources that could better support them and help buffer their care burden [41]. Their absence is especially alarming given both their increasing numbers and their higher levels of distress and burden in comparison to older adult child caregivers as well as other caregiver types in the family (e.g., spouses) [26,27,28]. This study elevates the importance of the young adult child caregiver’s voice while providing an innovative dyadic lens that demonstrates the importance of encompassing the perspective of both diagnosed mothers and their YACDs.

Collectively, the findings showcase the important role of mother–daughter communication in facilitating breast cancer caregiving and promoting better outcomes for both diagnosed mothers and their young adult daughters serving as their caregivers, thereby providing critical information for targeted mother–daughter interventions that could promote healthier outcomes for diagnosed mothers and YACDs. Results highlight how parent–child communication approaches (i.e., avoidance and openness) intersect with psychosocial outcomes like distress and relational satisfaction. As such, findings demonstrate that if we are to promote better biopsychosocial outcomes for diagnosed mothers and their caregiving daughters, we must recognize the influential role their communication plays in their well-being and attend to both relational partners’ needs.

While this study provides further evidence that parent–child-focused communication skills interventions are needed to promote healthy outcomes, findings also offer a psychosocial map for developing a targeted mother-YACD communication skills intervention. Diagnosed mothers and their YACDs described key communication skills for care interactions, while identifying the most challenging topics for them to navigate. Given YACDs are the most burdened and distressed family cancer caregiver [21,22,23,24,25,41,46,47,48,63], we explore the implications of the study findings in conjunction with providing a psychosocial guide for intervention development.

### 4.1. The Interrelated Nature of Mother–Daughter Communication and Psychosocial Well-Being

Findings demonstrate the interconnected experiences of diagnosed parents and their caregiving young adult children. Mothers and YACDs mirrored one another in what they perceive to be their most challenging cancer-related conversations. Reports validate previous studies on mothers’ communication with AYA daughters—that it is challenging to discuss mortality, negative emotions, and daughters’ risk [4,5,51]. YACDs and their mothers perceived that avoiding such topics would buffer each other from distress or burden—a common motivation driving mother–daughter communication after diagnosis [5,54].

At the same time, findings reveal distinct challenges that are potentially specific to when daughters are in young adulthood and in the caregiving role. For instance, both YACDs and diagnosed mothers reported avoiding caregiving burden discussions as well as conversations about relational issues. YACDs are known to struggle in their new role as caregiver this early in the lifespan and also report more burden than both older adult child caregivers and spousal caregivers [9,26,27,28]. It is likely that the relational shift of the daughter becoming the mother’s caregiver, particularly early in their adulthood as YACDs are just transitioning from adolescence, further challenges mother–YACD relational dynamics [77]. It is also noteworthy that YACDs and mothers identified treatment as a challenging topic. Treatment is typically more openly discussed among mothers and daughters across ages after diagnosis [5,54]. It is possible that treatment-related discussions are more challenging for caregiving daughter-mother relationships (as opposed to non-caregiving bonds), but also more stressful when in the treatment phase of the cancer continuum, as these mothers were.

Although both YACDs and mothers avoided such conversations to buffer each other from distress, their avoidant behavior was tied to worse outcomes, such as poorer relational satisfaction. Previous research on openness between mothers and daughters coping with breast cancer showed that diagnosed women, as well as survivors, with a more open mother–daughter communication pattern exhibit better relational satisfaction, whereas those engaged in avoidance have poorer physical health related to cancer (e.g., fatigue or pain) [54]. Thus, even though avoidance may be enacted with the intention of protecting each other, it may inadvertently contribute to distress during treatment and in survivorship. YACDs in this study did express frustration with their mothers’ avoidance, noting that it could inhibit their ability to provide care. At the same time, avoidant behavior may be driven by a YACD’s or mother’s distress state. This may be particularly true with regard to YACDs’ psychological state. Diagnosed mothers acknowledged struggling with topics as their YACDs appeared uncomfortable. The statistical findings further suggest that YACDs were more avoidant than their mothers on all of the cancer-related challenging topics.

Ultimately, diagnosed mothers’ and their YACDs’ psychological well-being (i.e., distress) may play a role in their ability to talk about cancer or, alternatively, their avoidant behavior may contribute to poorer psychosocial outcomes. Openness was linked to better outcomes for both mothers and YACDs. YACDs who were less avoidant of cancer discussions reported less distress and more vigor (i.e., a positive mood state that may also inform YACDs’ energy and ability to cope with burden). Mothers also reported better clinical communication skills when their YACDs perceived their relationship was characterized by openness. Moreover, YACDs’ and mothers’ avoidance was associated with more distress like anger, and mothers’ distress may be heightened, particularly during treatment, as they reported more negative mood states (i.e., distress) such as depression than YACDs. It is also possible that diagnosed mothers’ physical and psychological well-being are acutely interrelated during treatment. Additionally, it is likely that YACDs’ exacerbated stress and burden in their caregiver role may further contribute to their avoidance of challenging discussions. As YACDs themselves acknowledge, they may also avoid discussions to prioritize their mothers’ psychological well-being over their own [26,46]. Collectively, these findings further suggest the importance of a communication skills intervention, to provide them with strategic approaches that can promote healthier caregiving and coping experiences while reducing their avoidant behavior.

### 4.2. Navigating Challenging Breast Cancer Conversations: A Guide for Mothers and Daughters

While cancer talk and care conversations are challenging, learning to navigate such discussions is necessary to not only promote better health outcomes but also foster YACDs’ ability to provide care (and for their mothers to receive it). Research has demonstrated not only the importance of facilitating openness in cancer caregiving and coping, but also providing targeted guidance in line with developmental differences such as maturity level and sociohistorical differences in women’s preferences for disclosing health information [55]. The findings herein provide further direction for a communication skills intervention to help YACDs and their mothers talk about care-related issues in a manner that is health-promoting.

For example, both mothers and YACDs emphasized the need for open, honest communication, and mothers felt supported when YACDs were willing to talk. They also stressed the importance of staying positive, an approach preferred by non-caregiving AYA daughters and diagnosed mothers [55]. Both mothers and YACDs in our study also identified a new strategy—third-party involvement—which focused on receiving guidance from other sources on how to talk to each other. This strategy further indicates mothers’ and YACDs’ receptiveness to communication skills interventions (and clinicians’ guidance) as they themselves recognized they “really need a better way to communicate”. While communication skills interventions for adult child cancer caregivers are limited, our recent Healthy Communication Practice intervention, developed for midlife adult child caregivers of parents living with a blood cancer demonstrated that online, self-guided interventions are feasible and acceptable in promoting adult child caregivers’ clinical and family communication skill development, including navigating challenging conversations [73].

While findings further illustrate the care context (i.e., challenging conversations) in which they need help communicating (and thus, contexts for adapting the Healthy Communication Practice intervention for YACDs and mothers), results also suggest that when mothers and YACDs communicate more openly, they are less avoidant of even challenging topics. Both YACDs and mothers with relationships characterized by more openness were less avoidant of talking about their relationship issues. Furthermore, YACDs who reported more open mother–daughter communication were less avoidant of discussing their mother’s mortality whereas mothers reporting more open mother–daughter communication in their relationship were less avoidant of talking about care burden with their YACDs. These findings collectively support the notion that when diagnosed mothers and daughters have a relational history of being open, they are also less avoidant when facing cancer, even when discussing what they perceive to be the most challenging cancer topics. Helping diagnosed mothers and YACDs develop or hone their open communication skills through interventions can be key to reducing unhealthy avoidant behavior and ensuring they have critical care discussions.

### 4.3. Limitations & Future Directions

This is a challenging population to recruit, particularly dyads of YACDs and mothers. As such, the sample size was relatively low, and while there were interesting trends in analyses, a higher sample size could yield more significant findings, as well as the ability to examine actor-partner interaction effects. Additionally, all quantitative findings are correlational and measured at a single time point; thus, no causal inferences can be made. Most mothers and YACDs also identified as non-Hispanic white. Culturally targeted studies are needed to identify similarities and differences in communication challenges and preferred approaches that may be tied to culturally distinct family communication norms and expectations. YACDs were also mostly unmarried, in their early twenties, and part- or full-time students. Those YACDs with additional personal or family obligations (e.g., a spouse, children, having a career) likely encounter additional burdens and challenging dynamics. YACDs in the later range of young adulthood (e.g., 30–39) should be purposively sought in future studies to more fully represent the expansive lifespan phase of young adulthood.

## 5. Conclusions

Communication skills interventions for mothers and their YACDs can enhance YACDs’ ability to provide care and better ensure their mothers receive the support they need. Such resources can also concurrently promote healthier psychological and relational well-being for both diagnosed mothers and YACDs.

## Figures and Tables

**Table 1:** Participant demographics.

	Mothers	YACDs
Race	Asian = 3 Black = 5 Hispanic/Latina = 1 White = 32 White/Hispanic/Latina = 2	Asian = 1 Black = 4 Asian/Pacific Islander = 1 White = 26 White/Hispanic/Latina = 5
Ethnicity	Hispanic, Spanish, Latina = 11 Not Hispanic, Spanish, Latina = 32	Hispanic, Spanish, Latina = 12 Not Hispanic, Spanish, Latina = 27
Work Status	Not Currently Employed = 19 Employed Part Time = 8 Employed Full Time = 16	Not Currently Employed = 2 Employed Part Time = 5 Full Time Student, Not Working = 10 Part Time Student, Working = 6 Full Time Student, Working = 7
Education	High School Diploma or GED = 4 Some College = 3 2-Year College Degree = 7 4-Year College Degree = 13 Graduate Degree = 17	High School Diploma or GED = 5 Some College = 13 2-Year College Degree = 9 4-Year College Degree = 8 Graduate Degree = 4
Relationship	Single = 15 In a Long-Term Relationship = 28	Single = 20 In a Long-Term Relationship = 18
Income	Less than $25,000 = 6 $25,000–$50,000 = 4 $50,000–$75,000 = 4 $75,000–$100,000 = 5 $100,00–$125,000 = 4 $125,000–$150,000 = 20 More than $150,000 = 1	Less than $25,000 = 6 $25,000–$50,000 = 5 $50,000–$75,000 = 3 $75,000–$100,000 = 2 $100,00–$125,000 = 7 $125,000–$150,000 = 15 More than $150,000 = 1
Marital	Single/Never Married = 4 Widowed = 5 Married = 25 Separated = 2 Divorced = 7 Divorced, Remarried = 1	Single/Never Married = 35 Widowed = 1 Married = 3 Separated = 0 Divorced = 0 Divorced, Remarried = 0
Current Treatment	Chemotherapy = 13 Surgery = 5 Radiation = 15 Endocrine = 3	

Note: Content was generated by the authors.

**Table 2:** Descriptive statistics for mother and YACD topic avoidance.

Topic	Mother Mean (*SD*)	Daughter Mean (*SD*)
Death	3.23 (1.13)	3.56 (0.94) *
Treatment	2.55 (1.11)	2.38 (1.03)
Risk	2.37 (1.21)	2.30 (1.08)
Burden	2.89 (0.95)	2.87 (1.12)
Feelings	3.14 (0.88)	3.10 (1.10)
Relating	2.45 (1.07)	2.77 (1.06)
Healthcare	2.43 (1.05)	2.24 (0.97)

* Mean difference is significant, *p* < 0.05. Content was generated by the authors.

**Table 3:** Correlations between mother/YACD avoidant coping and topic avoidance.

	Death	Treatment	Risk	Burden	Feelings	Relating	Healthcare
Avoidant Coping (Mother)	0.43 **	0.53 **	0.19	0.49 **	0.33	0.21	0.56 **
Avoidant Coping (YACD)	0.38 *	0.65 **	0.42 **	0.53 **	0.54 **	0.33 *	0.49 **

* Correlation is significant, *p* < 0.05; ** correlation is significant, *p* < 0.01; content was generated by the authors.

**Table 4:** Correlations between mother’s distress and topic avoidance.

	Depression	Tension	Anger	Confusion	Fatigue	Vigor
Overall Topic Avoidance (Mother)	0.58 **	0.58 **	0.56 **	0.54 **	0.48 **	−0.28

** Correlation is significant, *p* < 0.01; content was generated by the authors.

**Table 5:** Correlations between YACD’s distress and topic avoidance.

	Depression	Tension	Anger	Confusion	Fatigue	Vigor
Overall Topic Avoidance (YACD)	0.28	0.15	0.46 **	0.21	0.31	−0.37 *

* Correlation is significant, *p* < 0.05; ** correlation is significant, *p* < 0.01; content was generated by the authors.

**Table 6:** Correlations between mother/YACD perceptions of openness and topic avoidance.

	Death	Treatment	Risk	Burden	Feelings	Relating	Healthcare
Openness (Mother)	−0.29	−0.31	0.02	−0.37 *	−0.28	−0.57 **	−0.17
Openness (YACD)	−0.33 *	0.01	0.16	−0.25	−0.20	−0.65 **	−0.10

* Correlation is significant, *p* < 0.05; ** correlation is significant, *p* < 0.01; content was generated by the authors.

## Data Availability

The datasets generated during and/or analyzed during the current study are available from the corresponding author upon reasonable request.
